# Longitudinal Symptom Burden Trajectories in a Population-Based Cohort of Women with Metastatic Breast Cancer: A Group-Based Trajectory Modeling Analysis

**DOI:** 10.3390/curroncol28010087

**Published:** 2021-02-14

**Authors:** Suman Budhwani, Rahim Moineddin, Walter P. Wodchis, Camilla Zimmermann, Doris Howell

**Affiliations:** 1 Institute for Health System Solutions and Virtual Care, Women’s College Hospital, Toronto, ON M5S 1B2, Canada; 2 Department of Family and Community Medicine, University of Toronto, Toronto, ON M5G 1V7, Canada; rahim.moineddin@utoronto.ca; 3 Institute of Clinical Evaluative Sciences, Toronto, ON M4N 3M5, Canada; walter.wodchis@utoronto.ca; 4 Institute of Health Policy, Management & Evaluation, University of Toronto, Toronto, ON M5T 3M6, Canada; 5 Health System Performance Network, University of Toronto, Toronto, ON M5T 3M6, Canada; 6 Institute for Better Health, Trillium Health Partners, Mississauga, ON L5M 2N1, Canada; 7 Princess Margaret Cancer Centre, University Health Network, Toronto, ON M5G 2C1, Canada; camilla.zimmermann@uhn.ca (C.Z.); Doris.Howell@uhn.ca (D.H.); 8 Department of Medicine, University of Toronto, Toronto, ON M5S 1A8, Canada; 9 Lawrence S. Bloomberg Faculty of Nursing, University of Toronto, Toronto, ON M5T 1P8, Canada

**Keywords:** breast neoplasms, neoplasm metastasis, palliative care, symptom assessment, syndrome, cohort studies, longitudinal studies, latent class analysis

## Abstract

Understanding the symptom burden trajectory for metastatic breast cancer patients can enable the provision of appropriate supportive care for symptom management. The aim of this study was to describe the longitudinal trajectories of symptom burden for metastatic breast cancer patients at the population-level. A cohort of 995 metastatic breast cancer patients with 16,146 Edmonton Symptom Assessment System (ESAS) assessments was constructed using linked population-level health administrative databases. The patient-reported ESAS total symptom distress score (TSDS) was studied over time using group-based trajectory modeling, and covariate influences on trajectory patterns were examined. Cohort patients experienced symptom burden that could be divided into six distinct trajectories. Patients experiencing a higher baseline TSDS were likely to be classified into trajectory groups with high, uncontrolled TSDS within the study follow-up period (χ^2^ (1, *N* = 995) = 136.25, *p* < 0.001). Compared to patients classified in the group trajectory with the highest relative TSDS (Group 6), patients classified in the lowest relative TSDS trajectory group (Group 1) were more likely to not have comorbidities (97.34% (for Groups 1–3) vs. 91.82% (for Group 6); *p* < 0.05), more likely to receive chemotherapy (86.52% vs. 80.50%; *p* < 0.05), and less likely to receive palliative care (52.81% vs. 79.25%; *p* < 0.0001). Receiving radiotherapy was a significant predictor of how symptom burden was experienced in all identified groups. Overall, metastatic breast cancer patients follow heterogeneous symptom burden trajectories over time, with some experiencing a higher, uncontrolled symptom burden. Understanding trajectories can assist in establishing risk-stratified care pathways for patients.

## 1. Introduction

In Canada, approximately 5% of all breast cancers in women were diagnosed as Stage IV between 2011 and 2015 [1]. Although constituting a smaller percentage of overall breast cancer diagnoses, Stage IV or metastatic breast cancer can pose a significant burden on both patients and the healthcare system [2,3,4,5,6]. Advances in treatment have resulted in a growing number of advanced cancer patients living longer [7,8,9,10]. Consequently, management of ongoing and long-term symptom burden from disease progression and treatment (chemotherapy, radiotherapy) by the patient, their families, healthcare providers and the healthcare system is essential [2,5,11,12]. While healthcare services such as home and palliative care can provide some support based on available capacity [13,14,15], advanced metastatic breast cancer can create significant challenges in the management of the disease and symptoms both for patients and providers, and result in unmet patient care needs over time [2,5,16,17,18,19,20]. As such, there is a need to better understand chronic, long-term patient symptom burden for metastatic breast cancer patients longitudinally. With this understanding, healthcare providers can better advise patients on appropriate treatment, provide supportive care such as self-management support and palliative care for patients earlier in the course of the disease trajectory, as well as create personalized and risk-stratified care pathways, thereby meeting patient and health system needs [21].

However, symptom burden in the literature has typically been examined longitudinally for cancer patients early in the course of the disease [22,23,24,25] or at the end of life [26,27,28,29,30]. Some studies have incorporated more extensive longitudinal measurement of symptom burden in patients at all stages of breast cancer, rather than focusing specifically on Stage IV metastatic breast cancer [6,31,32,33,34,35,36,37,38]. While some research has examined symptom burden longitudinally for patients with metastatic breast cancer, this research has been limited to a few assessments or time points of measurement of symptom burden [18,39,40], or has depicted average symptom burden across a largely heterogenous patient group [41]. Hence, the aim of this study was to identify and describe the longitudinal, heterogeneous trajectories of symptom burden in a population-based sample of metastatic breast cancer patients using data from multiple symptom burden assessments over time. A secondary aim was to examine the influence of covariates on symptom burden trajectory patterns.

## 2. Methods

### 2.1. Participants and Procedure

A longitudinal, observational approach was utilized, using retrospectively collected population-level administrative data from linked health administrative databases housed at Cancer Care Ontario in the province of Ontario, Canada. This study was approved by the University of Toronto Health Sciences Research Ethics Board, with patient consent considered implied due to the use of health administrative data.

From health administrative databases, a cohort of all adult (18 years of age or older at diagnosis), female patients diagnosed de novo with primary breast cancer and distant metastases (Stage IV) between 1 January 2010 and 31 December 2014 were identified using the Ontario Cancer Registry (OCR), a provincial registry of all residents with new diagnoses or deaths from cancer [42]. Stage IV metastatic breast cancer patients were identified using the tumour-node-metastasis (TNM) staging classification system and International Classification of Disease (ICD) codes utilized in the OCR. Individuals with any indication of multiple primary cancer diagnoses were excluded.

The constructed cohort of patients with Stage IV metastatic breast cancer was linked to the Interactive Symptom Assessment and Collection (ISAAC) database to obtain Edmonton Symptom Assessment System (ESAS) assessments for each patient from date of diagnosis until death or 31 December 2015, the end of the study follow-up period. The ESAS is a validated and reliable symptom screening tool for cancer patients [43,44] that assesses the severity of nine commonly experienced symptoms, namely pain, tiredness, drowsiness, nausea, lack of appetite, shortness of breath (dyspnea), depression, anxiety, and wellbeing on a scale of 0–10, with 0 indicating no symptoms (or best score for the symptom scale of wellbeing), and 10 serving as an indication of the most severe symptom experienced at a given point in time [44]. Patients complete the ESAS at each visit to an Ontario Cancer Centre and thus, data is collected mostly from ambulatory patients, with each patient potentially having multiple ESAS assessments longitudinally during the course of their care [45,46].

Inclusion and exclusion criteria for the study cohort were applied at the patient and ESAS assessment level. At the patient level, the following exclusions were made: (a) since longitudinal assessment of symptoms was required, 256 patients with fewer than three ESAS assessments were excluded from the study cohort; (b) six patients were found to have symptom scores of zero on all individual symptom scores for all collected ESAS assessments that after further examination of the dataset were assumed to be data error; these six patients were excluded from the study cohort, (c) three patients with missing values on the explanatory variable of neighbourhood income quintile were also excluded from the study cohort.

At the ESAS assessment level, a total of 58 ESAS assessments were excluded if more than 50% of the symptom scores on the assessment were missing. ESAS assessments with less than or equal to 50% of symptom scores missing in the assessment were used to impute total symptom distress score based on the mean of the non-missing scores available. ESAS assessments were also excluded from the cohort if patients had two ESAS assessments on the same day. In these cases, the ESAS assessment with the higher summative total symptom distress score (a sum of the nine individual scales comprising the ESAS) was included (n = 603 duplicate assessments excluded).

### 2.2. Measures

Total symptom distress score (TSDS) was the main outcome variable for this study, representing the symptom burden experienced by patients at a single time point of measurement [44]. It has been used previously in clinical trials to measure patient symptom severity [47,48]. TSDS scores were calculated by summing the nine ESAS physical and psychological symptom assessment scores from each unique ESAS assessment (pain, tiredness, drowsiness, nausea, appetite, dyspnea, anxiety, depression, and wellbeing) for each patient, providing a potential score of 0–90 for each assessment [44]. The TSDS did not follow a normal distribution; as such, a λ = 0.25 transformation was performed to normalize the TSDS using the Box–Cox transformation methodology to control for the skewness of data without losing the data’s heterogeneity [49,50,51], inclusive of a shift of +1 to ensure positive values. Transformed TSDS sores were analyzed and are reported.

Date of diagnosis until date of death or study endpoint for each patient were used to calculate months since diagnosis as the main time variable within the analysis. Time-fixed (baseline) covariates included age at diagnosis and year of diagnosis derived from the OCR; variables of neighbourhood rurality and neighbourhood income quintile were calculated using postal codes derived from linkage to the Registered Persons Database, which captures all registered Ontarians for publicly funded healthcare. Postal codes were linked to Statistics Canada census data to determine neighbourhood rurality and income quintile (a quintile of 1 having the lowest income, and of 5 having the highest income) [52,53,54]. Lastly, the Charlson Comorbidity Index, a measure of comorbidities, was derived from methodology based on patient hospitalization records [55,56,57] and excluding scoring for cancer and metastases [56,58].

To address temporality, we used time-varying covariates, which consisted of receipt of any treatment or supportive care 0–7 days prior to each ESAS assessment date. Treatment and supportive-care variables were selected based on their noted impact on symptoms for cancer patients in the literature [3,13,59,60,61]. Treatment variables included chemotherapy derived from the Activity Level Reporting System (ALR), and radiotherapy derived from the National Ambulatory Care Reporting System (NACRS) database, capturing all ambulatory and emergency department visits to hospitals by patients, and the ALR database. Supportive care included receipt of in-home, non-end-of-life home care services excluding case management, nurse practitioner palliative care, placement, and uncategorized ‘other’ services, derived from the Home Care Database (HCD). Supportive care also included receipt of palliative care services, which were identified based on a previously published methodology [15], including only services provided by physician providers using the Ontario Health Insurance Plan (OHIP) database and end-of-life home-care services from HCD excluding case management, placement, and uncategorized ‘other’ services. Finally, all emergency department visits for each patient were identified using the NACRS database. A rate of emergency department visits per person month was calculated.

### 2.3. Statistical Analysis

The statistical analysis focused on identifying the patterns and distinct number of longitudinal symptom trajectories of patients over time, using data collected through repeated measurement of patient symptom burden. For all individuals in the cohort, the first 30 ESAS assessments were selected for analysis due to the wide range of total number of ESAS assessments for each patient in the cohort. Standard growth models are useful to study variability over time through calculation of an average developmental trajectory, where all individuals in the study population are expected to change in the same direction and by the same strength over time. However, in certain variables such as symptom scores, patients may experience symptoms in different patterns over time, and capturing this variability becomes important. As such, the semi-parametric statistical technique of group-based trajectory modelling (GBTM) was selected and utilized for the purpose of identifying distinct subgroups of patients in the cohort following similar patterns of change over time on the outcome variable of TSDS [62,63,64,65,66]. The use of GBTM enables identification of distinct subgroups of patients following similar patterns of change over time, thereby improving estimates of experienced symptom burden (TSDS) by patients [64,66,67,68,69]. Trajectories are fitted using maximum-likelihood methods [64,69]. 

All analyses were performed using SAS v.9.4 software [70]. A SAS procedure to run the GBTM analysis was downloaded [71,72]. *p*-values less than 0.05 for two-sided tests were considered significant, and confidence intervals were calculated. An uncensored normal (CNORM) model was selected in the GBTM due to the continuous nature of the outcome variable of the TSDS, with minimum and maximum values specified outside the range of observed values due to no expected or demonstrated clustering [63,66,69]. Next, the number of groups and the order of polynomials defining the shape of the trajectory for each group as flat (0), linear (1), quadratic (2), cubic (3), or quartic (4) were iteratively fitted and specified. Due to limited a priori knowledge of the potential number of trajectory subgroups expected, the number of groups and each group’s polynomial order was decided by starting with a group number of one and highest polynomial order of quartic (4), and iterations were continued to find the optimal number of groups and polynomial order [64,66,73]. The optimal model was determined based on changes in Bayesian information criteria (BIC) values between models [69]. The BIC is a statistical measure of model fit based on assessment of increased model parameters balanced by increasing model complexity, with higher numbers indicating an improved model fit [64]. The BIC is used to approximate a second measure of model parsimony, the log of the Bayes factor, with >10 being considered strong evidence in favour of model parsimony [66,69,73]. However, in certain situations the BIC may not always clearly indicate the optimal number of groups and order of polynomials for each group, and in such cases the objective of seeking the greatest model parsimony should be balanced with the objective of reporting a representative number of distinctive trajectories from the data [64]. Other criteria considered in selecting the optimal model included the fit of the 95% confidence intervals to estimated trajectories and points of overlap [63], the statistical significance at a *p*-value of 0.05 for the highest-order standard coefficient in the results of the model, and the percentage of cohort in each group [66,71,74]. Following the selection of the optimal number of groups and polynomial order for each group, the final model fit was assessed using (a) average posterior probability (AvePP) (group assignment probability) >0.7, (b) odds of correct classification (OCC) >5 and (c) |π−Ρ| (estimated group probabilities versus the proportion of sample assigned to the group) ~ 0 [64,66]. A profile plot for each trajectory group in relation to covariates was created, and chi-square statistics or logistic regressions were conducted to explore whether patient demographic and clinical characteristics were associated with trajectory groups identified [64,68,75].

Finally, a three-stage process was followed to select baseline covariates to include in the above selected model as hypothesized predictors of group membership [64,68]. The first stage was the process by which the above unadjusted model was selected. In the second stage, a multivariable multinomial logistic model was run to examine the association between trajectory group assignment and time-fixed baseline covariates to understand baseline predictors of group membership. No statistically significant covariates at the *p* = 0.05 level were found. As such, no time-fixed baseline covariates were included in the re-estimation of trajectories in the third and final stage, to jointly estimate trajectories and associations between trajectories and covariates. Hence, only time-varying covariates (chemotherapy, radiotherapy, home care, and palliative care) were included in the estimation at this stage as predictors of the patterns of symptom burden for each group’s trajectory [64]. 

Two types of sensitivity analyses were performed. The first sensitivity analysis explored the relationship between each patient’s baseline median TSDS score and subsequent probability of being assigned to a higher symptom burden trajectory (Groups 4–6) or a changing, lower symptom burden trajectory group (Groups 1–3) over the disease trajectory, through calculation of chi-square statistics. The second sensitivity analysis assigned patients censored due to death in the follow-up time period with a maximum transformed TSDS to better understand the influence of death (or subsequent missing values of TSDS) on observed trajectories.

## 3. Results

**Figure 1:**
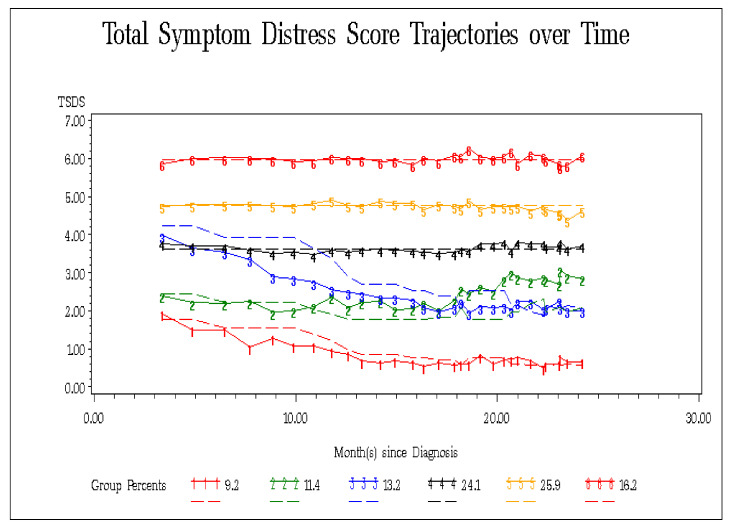
Total Symptom Distress Score (TSDS)* group trajectories over time. *Transformed values are reported. Magnitude of TSDS differences within group trajectories are relative, and not absolute values.

A total of 995 patients with 16,146 unique ESAS assessments were included in the study cohort (Table 1). Through iterative GBTM analysis, a six-group model with a polynomial order of 444000 was selected, demonstrating the lowest BIC value among all iterations and a log Bayes factor >100, indicating very strong evidence of model parsimony (Table 2). Figure 1 depicts the six trajectories for TSDS over time with percent group memberships for each group before adjustment with covariates. The 95% confidence intervals modelled for this trajectory demonstrated a close fit to the depicted trajectories and limited points of overlap, suggesting distinct trajectories (Supplementary Figure S1). Model-fit statistics demonstrated good model fit for the model selected: group membership was >5% for each group, AvePP for each group was above the recommended criteria of 0.7, the OCC was >5 for each group, and |π−Ρ| was also close to 0 for each group. Overall, trajectory Groups 4–6 demonstrated relatively higher, uncontrolled TSDS scores over time, while trajectory Groups 1–3 demonstrated changing symptom burden patterns over time and had relatively lower TSDS scores. The first sensitivity analysis indicated that for patients starting with a higher than median TSDS baseline score, there was an increased probability of being assigned to a high, uncontrolled TSDS group trajectory (Groups 4–6) in the follow-up time period (χ^2^ (1, *N* = 995) = 136.25, *p* < 0.001). The second sensitivity analysis demonstrated similar patterns as observed in Figure 1, other than for Groups 2 and 3.

Table 3 shows patient baseline characteristics by each of the six trajectory groups. Compared to individuals in Group 6 (the highest relative TSDS trajectory overall), individuals in Group 1 (the lowest relative TSDS trajectory overall) were more likely to not have any comorbidities (97.34% (for Groups 1–3) vs. 91.82% (for Group 6); *p* < 0.05), more likely to receive chemotherapy (86.52% vs. 80.50%; *p* < 0.05), and less likely to receive palliative care (52.81% vs. 79.25%; *p* < 0.0001). However, none of these baseline characteristics were significant in predicting a patient’s higher or lower symptom burden group membership. 

Table 4 describes parameter estimates for the original trajectory group model adjusted for time-varying covariates of group trajectory. This allowed us to determine how time-varying covariates of radiotherapy, chemotherapy, home care, and palliative care influenced the actual pattern or trajectory of symptom burden experienced by patients within each group. Receipt of radiotherapy was a significant predictor of TSDS trajectory patterns over time for all groups, while receipt of home care and palliative care each predicted TSDS patterns in four of the six groups. All covariates were significant predictors for the trajectory observed for Group 5. For Group 3, which showed a trajectory of most relative improvement in TSDS over time, radiotherapy and palliative care were both significant predictors, while for Group 2, which showed a trajectory of relative increase in TSDS over time, chemotherapy and radiotherapy were significant predictors.

Table 5 shows the clinical parameters for the adjusted group membership. Lower percentages of patients in Groups 1 to 3 (lower TSDS) received chemotherapy and palliative care in comparison to Groups 4 to 6 (higher TSDS). Groups 2 and 3 also generally had lower percentages of patients with death within three years of diagnosis in comparison to Groups 4 to 6.

## 4. Discussion

This study characterized the progression of symptom burden in a population-based cohort of women with advanced breast cancer based on the TSDS from ESAS assessments conducted over the disease trajectory. Six distinct symptom burden trajectories were identified for this population group that can be further grouped into a low symptom burden and changing trajectory over time (Groups 1 to 3) and a high, uncontrolled symptom burden trajectory over time (Groups 4 to 6). These results are similar to those observed in other studies that demonstrated differences in how patients with breast cancer experience symptom burden [31,33]. The identification of two subgroups of cancer patients, one experiencing low symptom burden and the other experiencing relatively higher symptom burden, has also been found in other studies of different cancer populations [25,26]. Findings from this study reiterate these findings specifically for the patient population of advanced metastatic breast cancer, suggesting similarities across diverse cancer population groups.

Our analysis also showed that there were no baseline demographic and clinical characteristics that were associated with a patient’s membership in a particular trajectory group; however, the symptom burden trajectory for patients in each group was influenced by covariates of chemotherapy, radiotherapy, home care, and palliative care in different ways. Our analysis demonstrates that demographic and clinical characteristics, such as comorbidities, do not have an influence on how patients experience symptom burden, potentially suggesting the independent effect of treatment on symptom burden experienced. However, this might also be because demographic and clinical characteristics, such as functional status and disease severity, were not included in the analysis as they could not be derived from our utilized data sources. Moreover, while home care and palliative care were modelled in this analysis as predictors of symptom burden, they are likely proxies for symptom burden, that is the reason why patients may be eligible for home care and palliative care due to high symptom burden.

Our study also found that patients in Groups 4 to 6 had relatively higher symptom scores and an uncontrolled symptom burden trajectory over time. This might be because these patients may be on a closer trajectory towards death, as was also demonstrated by a larger percentage of patients dying within the first three years of diagnosis in Groups 4 to 6 (all high, uncontrolled symptom trajectory groups). Additionally, a greater proportion of patients in these groups also resided in lower income and urban neighbourhoods, suggesting possible challenges to accessing breast cancer screening and a cancer diagnosis [76,77]. Findings from the sensitivity analysis demonstrated that patients with a TSDS higher than or equivalent to the median at diagnosis, were more likely to follow a high, uncontrolled symptom burden trajectory over time, a finding also demonstrated in other studies [29,31]. This demonstrates the importance of the assessment of symptom burden from point of diagnosis and the need for appropriate symptom management and supportive care, such as palliative care and home care from disease onset, particularly for individuals with a high, uncontrolled symptom burden. Moreover, this study found that some patients have changing (increasing or decreasing) symptom burden over time (Groups 1 to 3), a finding also demonstrated by Kang and colleagues for the general cancer population [29]. Groups 1 to 3 had a larger percentage of patients who received chemotherapy in comparison to other groups, suggesting receipt of active treatment may be the reason for changing symptom trajectories over time [78]. This subset of patients would benefit from appropriate supportive care, such as symptom self-management strategies to control symptom burden associated with active treatment over time. Overall, these results suggest that there are sub-sets of patients that may benefit from proactive symptom-support intensification and help set the foundational groundwork for the creation of personalized risk-stratified cancer care pathways [21].

While this study has shown distinct trajectories of symptom burden in women with metastatic breast cancer, there are some limitations that must be considered. First, while distinct trajectories were estimated for this cohort, they should not be considered reified groups or absolute certainties [64,73]. The use of administrative data precluded examination of demographic and clinical characteristics such as functional status, specific sites of metastasis, biological subtype of breast cancer, and disease severity, which may influence symptom trajectory patterns [36,41]. Moreover, certain symptom scores on the ESAS may not be as sensitive to changes in symptom burden over time (e.g., appetite) [79]. In addition, model selection, although based on model fit statistics indicating parsimony, was also based on other recommended criteria requiring judgment. This is inherently a part of GBTM [64], thus presenting a need for more specific delineation of the model selection process in the literature [80]. Lastly, this study examined TSDS, which may mask a symptom score change on single symptoms over time [81]. Future research examining individual symptom score trajectory patterns over time, particularly differences in physical and psychosocial symptoms, will be important for identifying populations at risk who may require intensification of symptom management for specific symptoms.

## 5. Conclusions

Overall, this study provides important findings by identifying the nuances of symptom burden trajectories experienced by metastatic breast cancer patients; the relative contribution of chemotherapy, radiotherapy, home care, or supportive care on experienced symptom burden; and the importance of focusing attention on patients who are diagnosed with high symptom burden. These patients may potentially live with high symptom burden for the duration of the disease with the assistance of targeted palliative care and self-management support strategies and education via a personalized, risk-stratified care delivery approach. Further assessment by healthcare providers of patient symptom burden (high/uncontrolled or low/changing over time) would facilitate symptom control in this population of metastatic breast cancer patients with advanced disease who are in a dual state of survivorship and limited mortality [16,82,83]. This study helps set the foundational groundwork for the creation of personalized risk-stratified cancer-care pathways considered critical for cancer care in the future [21]. Future research can build on this research to examine other factors that were not able to be examined in this study, such as disease severity, functional status, and caregiver burden, among others [21], and their role in and contribution to patient symptom burden trajectories over time. Future research can also examine potential variations in trajectories of individual symptom scores for patients [81]. This will enhance the understanding of symptom burden over time in the metastatic breast cancer patient population leading to better symptom burden control, symptom management, and improved quality of life for patients.

## Figures and Tables

**Table 1:** ESAS assessments per patient.

	All Patients in Cohort (n = 995)	
	n	%
Age Group ^a^		
18–39	71	7.14%
40–49	172	17.29%
50–59	254	25.53%
60–69	241	24.22%
70–79	170	17.09%
80–99	87	8.74%
Neighborhood Income Quintile ^a^		
Quintile 1 (Low Income)	195	19.60%
Quintile 2	196	19.70%
Quintile 3	193	19.40%
Quintile 4	212	21.31%
Quintile 5 (High Income)	199	20.00%
Rurality ^a^		
Urban	880	88.44%
Rural	115	11.56%
Diagnosis Year		
2010	196	19.70%
2011	194	19.50%
2012	204	20.50%
2013	213	21.41%
2014	188	18.89%
Charlson Comorbidity Index ^a^		
0	949	95.38%
1–3	46	4.62%
Receiving Treatment—Chemotherapy ^b^		
No	149	14.97%
Yes	846	85.03%
Receiving Treatment—Radiotherapy ^b^		
No	544	54.67%
Yes	451	45.33%
Receiving Care—Home Care ^b^		
No	485	48.74%
Yes	510	51.26%
Receiving Care—Palliative Care ^b^		
No	307	30.85%
Yes	688	69.15%
Survival Time		
Between 0 to 1 Year	135	13.57%
Between 1 to 3 Years	275	27.64%
Between 3 to 5 Years	77	7.74%
Greater than 5 Years	9	0.90%
Censored ^c^	499	50.15%
ESAS Scores Baseline ^d,e^		
Mean Total Symptom Distress Score (SD, Range, N)	20.33 (15.73, 0.00–79.00, 995)	
Mean Pain Score (SD, Range, N)	2.33 (2.63, 0.00–10.00, 992)	
Mean Tiredness Score (SD, Range, N)	0.86 (1.84, 0.00–10.00, 995)	
Mean Lack of Appetite Score (SD, Range, N)	1.79 (2.44, 0.00–10.00, 994)	
Mean Shortness of Breath Score (SD, Range, N)	1.47 (2.37, 0.00–10.00, 993)	
Mean Nausea Score (SD, Range, N)	3.45 (2.81, 0.00–10.00, 995)	
Mean Drowsiness Score (SD, Range, N)	2.44 (2.85, 0.00–10.00, 991)	
Mean Depression Score (SD, Range, N)	1.75 (2.51, 0.00–10.00, 995)	
Mean Anxiety Score (SD, Range, N)	3.04 (2.90, 0.00–10.00, 993)	
Mean Wellbeing Score (SD, Range, N)	3.21 (2.69, 0.00–10.00, 990)	
ED Visits ^f^		
Rate of ED Visits per Patient Month (Standard Error, Confidence Interval)	0.12 (0.02, 0.12–0.12)	

^a^. Baseline. ^b^. 0–7 days prior on any of 30 ESAS assessments. ^c^. Censored as at end of follow-up time period of December 31, 2015. ^d^. Untransformed values. ^e^. N for symptom scores may differ from sample cohort due to missing symptom scores on some assessments. ^f^. Patient month is calculated as total number of months patient was observed in study period (not limited to period of first 30 ESAS assessments). SD = Standard Deviation, ED = Emergency Department.

**Table 2:** Summary of model selection results.

Number of Groups	Polynomial Order	BIC ^a^	log Bayes Factor ^b^
1	4	−33,389.92	-
2	44	−29,166.78	>1000
3	444	−27,620.32	>1000
4	4440	−27,075.20	>1000
5	44401	−26,746.86	>100
6	444000	−26,486.85	>100

^a^. BIC=Bayesian information criteria. Model with lowest BIC value is preferred. ^b^. A log Bayes factor of >10 indicates very strong evidence of model parsimony.

**Table 3:** Patient characteristics by trajectory group (unadjusted).

	All Patients in Cohort											
	Group 1		Group 2		Group 3		Group 4		Group 5		Group 6	
Total N	89		138		111		233		265		159	
	n	%	n	%	n	%	n	%	n	%	n	%
Age Group ^a,b^												
18–39	7	7.87%	13	9.42%	10	9.01%	19	8.15%	14	5.28%	8	5.03%
40–49	14	15.73%	20	14.49%	23	20.72%	43	18.45%	40	15.09%	32	20.13%
50–59	25	28.09%	28	20.29%	30	27.03%	61	26.18%	75	28.30%	35	22.01%
60–69	19	21.35%	39	28.26%	26	23.42%	61	26.18%	64	24.15%	32	20.13%
70–79	18	20.22%	28	20.29%	16	14.41%	34	14.59%	41	15.47%	33	20.75%
80–99	6	6.74%	10	7.25%	6	5.41%	15	6.44%	31	11.70%	19	11.95%
Neighbourhood Income ^a^												
Quintile 1 (Low Income)	19	21.35%	27	19.57%	12	10.81%	45	19.31%	53	20.00%	39	24.53%
Quintile 2	11	12.36%	23	16.67%	25	22.52%	51	21.89%	55	20.75%	31	19.50%
Quintile 3	20	22.47%	28	20.29%	19	17.12%	48	20.60%	52	19.62%	26	16.35%
Quintile 4	23	25.84%	30	21.74%	28	25.23%	47	20.17%	47	17.74%	37	23.27%
Quintile 5 (High Income)	16	17.98%	30	21.74%	27	24.32%	42	18.03%	58	21.89%	26	16.35%
Rurality ^a^												
Urban	74	83.15%	123	89.13%	99	89.19%	207	88.84%	230	86.79%	147	92.45%
Rural	15	16.85%	15	10.87%	12	10.81%	26	11.16%	35	13.21%	12	7.55%
Diagnosis Year ^a^												
2010	15	16.85%	25	18.12%	21	18.92%	46	19.74%	51	19.25%	38	23.90%
2011	19	21.35%	20	14.49%	20	18.02%	50	21.46%	53	20.00%	32	20.13%
2012	16	17.98%	23	16.67%	26	23.42%	51	21.89%	55	20.75%	33	20.75%
2013	21	23.60%	30	21.74%	23	20.72%	49	21.03%	59	22.26%	31	19.50%
2014	18	20.22%	40	28.99%	21	18.92%	37	15.88%	47	17.74%	25	15.72%
Charlson Comorbidity Index ^c,d^												
0	329			97.34%			227	97.42%	247	93.21%	146	91.82%
1–3	9			2.66%			6	2.58%	18	6.79%	13	8.18%
Receiving Treatment—Chemotherapy ^b,d^												
No	12	13.48%	17	12.32%	9	8.11%	28	12.02%	52	19.62%	31	19.50%
Yes	77	86.52%	121	87.68%	102	91.89%	205	87.98%	213	80.38%	128	80.50%
Receiving Treatment—Radiotherapy ^b^												
No	53	59.55%	85	61.59%	61	54.95%	120	51.50%	141	53.21%	84	52.83%
Yes	36	40.45%	53	38.41%	50	45.05%	113	48.50%	124	46.79%	75	47.17%
Receiving Care—Home Care ^b^												
No	47	52.81%	71	51.45%	53	47.75%	105	45.06%	133	50.19%	76	47.80%
Yes	42	47.19%	67	48.55%	58	52.25%	128	54.94%	132	49.81%	83	52.20%
Receiving Care—Palliative Care ^b,d^												
No	42	47.19%	51	36.96%	41	36.94%	73	31.33%	67	25.28%	33	20.75%
Yes	47	52.81%	87	63.04%	70	63.06%	160	68.67%	198	74.72%	126	79.25%
ER Visits/Person Month ^e^												
Mean ER Visits/Person Month	0.10		0.10		0.08		0.12		0.14		0.17	

^a^. Baseline ^b^. On first 30 ESAS assessments following diagnosis ^c^. Data for Groups 1–3 have been merged due to small cell numbers ^d^. Bivariate relationship between group and covariate significant at *p* = 0.05 level ^e^. Bivariate relationship not examined.

**Table 4:** Parameter estimates, errors, tests, and *p*-values for adjusted model.

Group Number (Membership %)	Parameter	Estimate	Standard Error	Test	*p*-Value
1 (11.49%)	Chemotherapy	−0.10	0.06	−1.67	0.0944
	Radiotherapy *	0.60	0.13	4.71	<0.0001
	Home Care *	0.35	0.09	3.82	0.0001
	Palliative Care *	0.38	0.07	5.35	<0.0001
2 (9.86%)	Chemotherapy *	−0.15	0.06	−2.66	0.0079
	Radiotherapy *	0.43	0.11	3.76	0.0002
	Home Care	0.11	0.08	1.47	0.1412
	Palliative Care	0.12	0.06	1.89	0.0592
3 (12.10%)	Chemotherapy	−0.06	0.06	−1.10	0.2734
	Radiotherapy *	0.43	0.11	4.01	0.0001
	Home Care	0.02	0.10	0.19	0.8466
	Palliative Care *	0.37	0.06	5.68	<0.0001
4 (24.63%)	Chemotherapy	−0.07	0.04	−1.74	0.0822
	Radiotherapy *	0.25	0.08	3.36	0.0008
	Home Care *	0.17	0.06	2.81	0.0050
	Palliative Care *	0.34	0.05	7.47	<0.0001
5 (26.48%)	Chemotherapy *	−0.16	0.04	−3.80	0.0001
	Radiotherapy *	0.24	0.08	3.02	0.0025
	Home Care *	0.22	0.06	3.92	0.0001
	Palliative Care *	0.15	0.04	3.43	0.0006
6 (15.44%)	Chemotherapy	0.01	0.05	0.18	0.8568
	Radiotherapy *	0.30	0.11	2.83	0.0047
	Home Care *	−0.14	0.07	−2.02	0.0430
	Palliative Care	−0.004	0.06	−0.07	0.9469

* Significant at *p* = 0.05.

**Table 5:** Clinical parameters by trajectory group for adjusted model.

	Group 1 (n = 121)	Group 2 (n = 93)	Group 3 (n = 114)	Group 4 (n = 245)	Group 5 (n = 271)	Group 6 (n = 151)
Chemotherapy (Y)	88.43%	86.02%	87.72%	88.98%	81.18%	80.13%
Radiotherapy (Y)	36.36%	43.01%	47.37%	48.16%	46.86%	45.03%
Home Care (Y)	52.89%	49.46%	50.88%	53.06%	49.45%	51.66%
Palliative Care (Y)	66.12%	58.06%	62.28%	66.53%	74.17%	78.81%
Radiotherapy + Chemotherapy (Y)	33.88%	36.56%	40.35%	42.45%	37.64%	38.41%
Radiotherapy + Home Care (Y)	23.14%	20.43%	25.44%	26.94%	25.09%	25.17%
Radiotherapy + Palliative Care (Y)	25.62%	24.73%	32.46%	35.1%	35.06%	37.09%
Home Care + Chemotherapy (Y)	50.41%	45.16%	46.49%	48.16%	43.17%	42.38%
Home Care + Palliative Care (Y)	38.84%	32.26%	33.33%	37.55%	39.85%	41.06%
Palliative Care + Chemotherapy (Y)	60.33%	52.69%	54.39%	61.22%	62.73%	62.91%
Chemotherapy + Radiotherapy + Home Care + Palliative Care (Y)	17.36%	12.90%	15.79%	17.96%	16.97%	18.54%
Time to Death <= 3 Years	44.63%	19.35%	20.18%	41.22%	47.23%	56.95%
Time to Death >3 Years	5.79%	15.05%	10.53%	8.98%	6.64%	8.61%

## Supplementary Materials

The following are available online at https://www.mdpi.com/1718-7729/28/1/87/s1: Supplementary Figure S1—Total Symptom Distress Score Trajectories over Time (95% Confidence Intervals)
