# Supplementary material for: Longitudinal Symptom Burden Trajectories in a Population-Based Cohort of Women with Metastatic Breast Cancer: A Group-Based Trajectory Modeling Analysis
Source: Curr Oncol. 2021 Feb 14;28(1):879–97. doi: 10.3390/curroncol28010087 (PMC7985757; doi:10.3390/curroncol28010087)
Supplement: Supplementary file 1 [file curroncol-28-00087-s001.pdf]

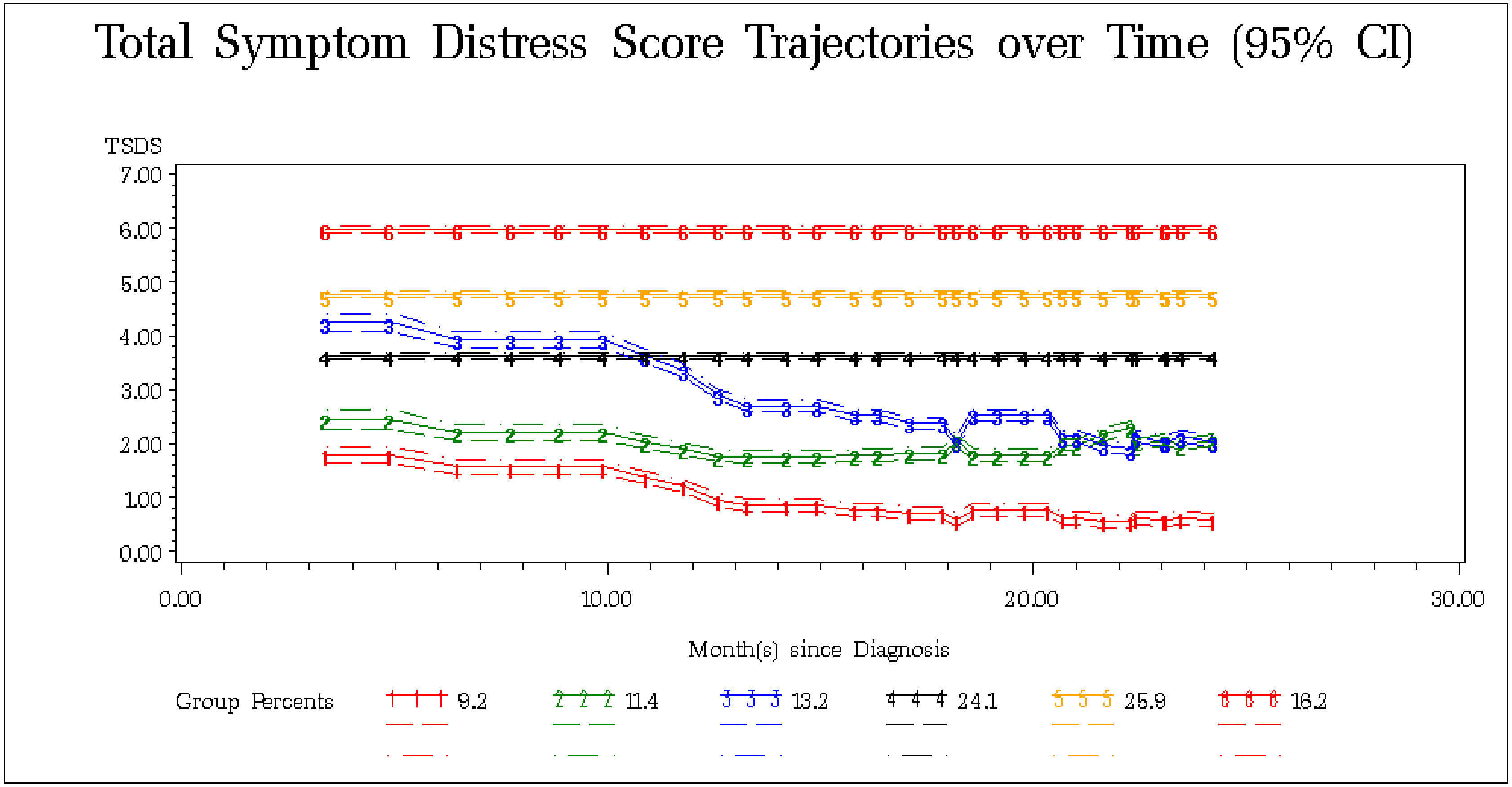

Supplementary Figure 1: Total Symptom Distress Score (TSDS)* group trajectories over time with 95% Confidence Intervals. *Transformed values are reported. Magnitude of TSDS differences within group trajectories are relative, and not absolute values.
